# Development from recurrent anti-*N*-methyl-*D*-aspartate receptor encephalitis with seizures as the first symptom to autoimmune-associated epilepsy: a case report

**DOI:** 10.1186/s42494-023-00129-0

**Published:** 2023-07-26

**Authors:** Ningxiang Qin, Jing Wang, Xi Peng, Liang Wang

**Affiliations:** 1 Department of Neurology, The First Affiliated Hospital of Chongqing Medical University, Chongqing, 400016 China; 2 Department of Neurology, The Second Affiliated Hospital of Chongqing Medical University, Chongqing, 400010 China

**Keywords:** Anti-*N*-methyl-*D*-aspartate receptor encephalitis, Autoimmune-associated epilepsy, Seizure, Relapse, Case report

## Abstract

**Background:**

Anti-*N*-methyl-*D*-aspartate receptor (anti-NMDAR) encephalitis is a novel autoimmune encephalitis (AE) first identified in 2007. It provides a new direction for clinicians when encountering unexplained symptoms such as seizures, psychotic behavioral abnormalities, speech disorders, and involuntary movements. Most patients have a good prognosis after immunotherapy, but some may experience relapses.

**Case presentation:**

We report a Chinese female patient diagnosed with anti-NMDAR encephalitis. Over the past 30 years, the patient had experienced eight episodes with seizures as the first symptom, which eventually progressed to autoimmune-associated epilepsy. In the last two episodes, both serum and cerebrospinal fluid of the patient were negative for AE-related antibodies, and brain magnetic resonance imaging (MRI) revealed abnormal hyperintensity in the bilateral hippocampi. The patient's symptoms were poorly controlled by immunotherapy but well controlled by anti-seizure medicines.

**Conclusions:**

Patients with a long history of AE and multiple relapses that start with seizures may display alterations of brain structure. Physicians should pay attention to autoimmune-associated epilepsy.

## Background

Anti-*N*-methyl-*D*-aspartate receptor (anti-NMDAR) encephalitis is a type of autoimmune encephalitis (AE) with clinical features such as behavioral/psychiatric problems, cognitive abnormalities, seizures, movement disorders, involuntary movement, autonomic instability, and central hypoventilation [1]. More than half of individuals with anti-NMDAR encephalitis develop convulsions, which are a common symptom [2]. Currently, immunological factors are a contributing component in epilepsy, and some researchers have found an association between autoimmune factors and a particular group epilepsies with unknown causes [3]. Thus, anti-NMDAR encephalitis with seizures as the first symptom may be confused with autoimmune-associated epilepsy. In July 2020, the International League Against Epilepsy (ILAE) proposed two conceptual definitions: “acute symptomatic seizures secondary to AE” and “autoimmune-associated epilepsy”, providing clinicians with some new ideas [4]. Here, we describe a case of anti-NMDAR encephalitis with symptoms progressing from acute symptomatic seizures secondary to AE to autoimmune-associated epilepsy.

## Case presentation

**Fig. 1:**
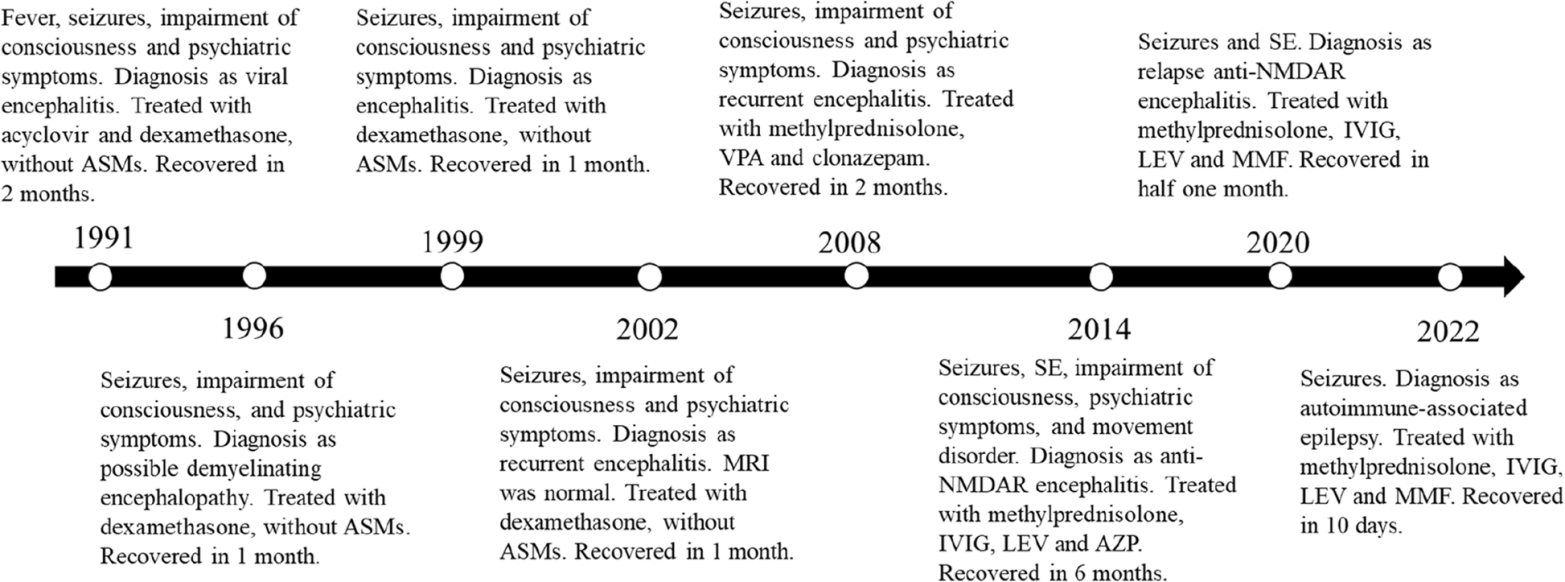
Timeline of the eight episodes of the patient, as well as the clinical presentation, treatment, and prognosis at each episode. ASM: anti-seizure medication; VPA: sodium valproate; AZP: azathioprine; LEV: levetiracetam; MMF: mycophenolate mofetil

**Fig. 2:**
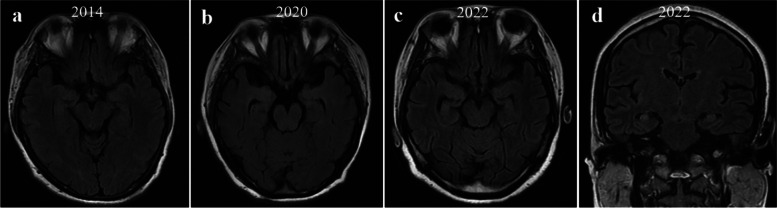
Brain MRI images of the patient in 2014, 2020, and 2022. **a, b** Brain MRI was normal, and bilateral hippocampal volume was normal in the cross-sectional images in 2014 (**a**) and 2020 (**b**). (**c**) Bilateral hippocampal volume was decreased in the cross-sectional images in 2022. **d** Brain MRI showed suspicious hyperintensity in the bilateral hippocampi in the coronal image in 2022

**Fig. 3:**
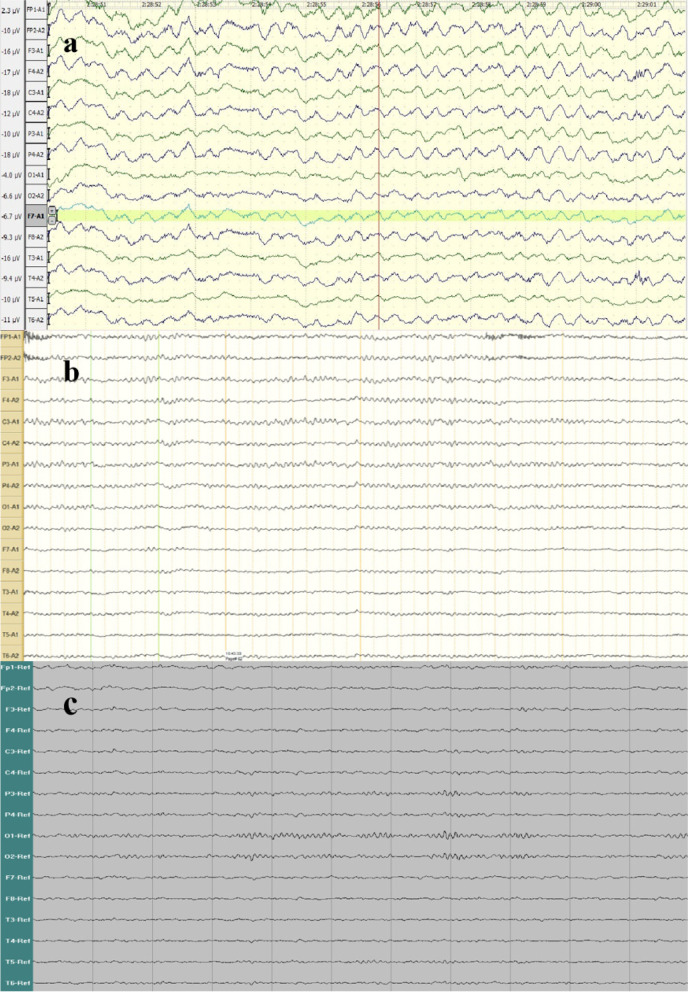
EEG recordings in 2014, 2020, and 2022. EEG demonstrated diffuse low- to high-amplitude slow waves in 2014 (**a**), and normal activities in 2020 (**a**) and 2022 (**c**)

A 35-year-old female was admitted on January 21, 2014 in the First Affiliated Hospital of Chongqing Medical University, with an over 20-year history of recurrent generalized tonic‒clonic seizures and psychiatric symptoms and 1 + month of recurrence. Notably, she had similar symptoms in 1991, 1996, 1999, 2002, and 2008, each recovered after 2–6 months of short-term glucocorticoid therapy (Fig. 1). She only exhibited seizures and psychiatric symptoms during the acute phase and the symptoms disappeared during the recovery phase. The patient took sodium valproate 500 mg bid regularly during the past 6 years, without occurrence of seizures, and was completely normal during interphases. After admission, levetiracetam 500 mg bid was given, but with unsuccessful seizure control. Over the next 10 days, she displayed status epilepticus (SE), screaming, resistant eye opening, no response to painful stimuli, catatonia, orofacial lingual dyskinesia, stiffness and autonomic manifestations, including hyperthermia, tachycardia, and hypertension. She required breathing support due to hypoventilation and was treated in the intensive care unit (ICU). The patient showed no focal neurological deficits except suspected neck rigidity. Cerebral spinal fluid (CSF) analysis demonstrated leucocytosis (12 × 10^6^/l, reference range < 8 × 10^6^/l), elevated protein level (0.83 g/l, reference range < 0.6 g/l), and negative viral infection. Brain magnetic resonance imaging (MRI) was normal (Fig. 2a). Electroencephalography (EEG) recording demonstrated diffuse low- to high-amplitude slow waves during a period with no seizure attack (Fig. 3a). Whole-body CT scan did not show presence of tumors, including teratomas. Both CSF and serum were positive for anti-NMDAR antibodies. Thus, the patient was diagnosed with anti-NMDAR encephalitis and given intravenous immunoglobulin (IVIG) and glucocorticoids for treatment. After 6 months, she progressively recovered with no epileptic seizures and no involuntary movement. After discharge, she received levetiracetam 500 mg bid and azathioprine 50 mg bid orally to prevent relapse.

On February 10, 2020, she experienced a seizure attack and was admitted to the hospital again. After admission, she was given IVIG and a pulse glucocorticoid therapy, but still had cluster seizures and developed SE. Then, she was transferred to the ICU. CSF analysis showed a CSF pressure of 170 mmH_2_O, and normal protein level (0.19 g/l) with pleocytosis (total cell count 51 × 10^6^/l, nucleated cell count 10 × 10^6^/l). AE-related antibodies were not detected in the serum and the CSF. Chest and whole-abdomen CT scans did not reveal tumors. Brain MRI and EEG recording showed normal results (Figs. 2b and 3b). Levetiracetam was given to control seizures, and diazepam and midazolam were given to manage SE. The patient gradually recovered and was prescribed mycophenolate and levetiracetam 500 mg bid after discharge.

On October 4, 2022, she had a seizure attack and was readmitted to our hospital. Interestingly, results of interictal EEG (Fig. 3c) and CSF test were normal. Both serum and CSF were negative for AE-related antibodies. However, brain MRI showed suspicious hyperintensity in the bilateral hippocampi (Fig. 2c and d). She did not experience psychiatric symptoms after being admitted, nor did she have a further seizure attack. She was given levetiracetam 500 mg bid. After 10 days of hospitalization, she was discharged and prescribed levetiracetam 500 mg bid and mycophenolate mofetil 500 mg bid after discharge.

## Discussion

The patient presented typical clinical manifestations of anti-NMDAR encephalitis in 2014 and before, including seizures, psychosis, abnormal movements, and autonomic and breathing instability, and the diagnosis was confirmed by the NMDAR antibody test in 2014. The symptoms of later attacks and the treatment response were very similar to what was observed in 2014. We therefore propose that the repeated events were actually relapses. Notably, although relapse is an important characteristic of anti-NMDAR encephalitis, this case is different from common anti-NMDAR encephalitis. As of 2014, this patient already had a history of over 20 years, with 6 attacks with seizures as the first symptom, a frequency higher than that reported in the literature. A cohort study of 244 AE patients from western China showed that 15.9% of the patients had one or multiple relapses, with 82.0% experiencing the first relapse within 24 months [5]. Zhong et al. conducted a retrospective review comprising 100 cases and revealed that the general relapse rate of anti-NMDAR, anti-GABABR, and anti-LGI1 encephalitis is 26% [6]. But notably, Liu et al. [7]. reported that female sex may also be a risk factor for seizure recurrence, but the precise mechanism is yet unknown. And some studies have shown that early immunotherapy can shorten the time to seizure cessation [8], and the risk of subsequent relapse is increased in those with delayed immunotherapy in the first episode [5, 6, 9]. For this patient, the multiple relapses may be related to delayed initial diagnosis and immunotherapy. The presence of AE-related antibodies in the patient's CSF or serum was not tested before admission to our hospital (before 2014). Thus, during the course of more than 20 years, the patient did not receive a definitive diagnosis or systematic treatment. Currently, the recommended first-line immunotherapies for patients with AE include intravenous steroids, IVIG, and plasma exchange. Although this patient had received short-term glucocorticoid therapy and some episodes had been controlled, she did not receive a powerful immunotherapy regimen for AE.

The patient was admitted to our hospital twice (in February 2020 and October 2022). These two episodes were different from previous ones in several aspects. First, the patient's symptoms were different from those in 2014 and earlier, in that the epileptic seizures were the main manifestation in the recent two episodes. Second, both serum and CSF were negative for AE-related antibodies at admission. Third, in the most recent two episodes, the patient had poor response to immunotherapy (IVIG and the pulse methylprednisolone) and even developed to SE. The main treatment strategy was based on anti-seizure medications. According to the new concepts proposed by ILAE in 2020, we defined the seizures that occurred in 2014 and before as acute symptomatic seizures secondary to AE. However, the last two episodes suggest that the condition may have progressed to autoimmune-associated epilepsy [4]. Liu et al. [7] reported that a small proportion of patients with AE have seizure recurrences, and 3.1% would develop chronic epilepsy during prolonged follow-up. For autoimmune-associated epilepsy, ILAE suggest that epilepsy may result from ongoing brain autoimmunity and from associated structural abnormalities of the brain. The patient had an approximately 30-year history of recurrent AE with seizures as the first symptom. In addition, the latest brain MRI examination showed suspicious hyperintensity in the bilateral hippocampi, suggesting the possibility of structural changes in the brain. All of these fit the new definition of the etiology of autoimmune-associated epilepsy. Of course, not all patients with chronic epilepsy have MRI or histopathological evidence of hippocampal atrophy. Neuroimaging may not always be able to indicate microscopic structural alterations in the brain [10]. In addition, it was noteworthy that although both serum and CSF were negative for AE-related antibodies at the last two admissions, we still considered the diagnosis of autoimmune-associated epilepsy based on the patient's long medical history and each episode. In most cases, the diagnosis of autoimmune-associated epilepsy should be based on a combination of clinical characteristics, MRI results, and CSF analysis [10]. The poor outcome of immunotherapy in the patient is consistent with the findings of a recent report by the International League Against Epilepsy (ILAE) in 2020, which highlights the limited effectiveness of immunotherapy in patients with autoimmune epilepsy [4]. In addition, antibody-negative patients are reported to have a higher rate of SE episodes, which are typically associated with a worse prognosis [10]. Ilyas-Feldmann et al. [11] revealed that delayed immunotherapy is also associated with the development of drug-resistant epilepsy.

## Conclusions

The patient presented here experienced disease progression from recurrent anti-NMDAR encephalitis with seizures as the first symptom to autoimmune-associated epilepsy. It contributes to the understanding of acute symptomatic seizures secondary to autoimmune encephalitis and autoimmune epilepsy arising from this case.

## Data Availability

The datasets generated during the current study are available from the corresponding author on reasonable request.

## Abbreviations

AE: Autoimmune encephalitis
Anti-NMDAR: Anti-*N*-methyl-*D*-aspartate receptor
CSF: Cerebrospinal fluid
EEG: Electroencephalography
ILAE: The International League Against Epilepsy
IVIG: Intravenous immunoglobulin
MRI: Magnetic resonance imaging
